# Targeting the Glutamatergic System to Treat Pathological Gambling: Current Evidence and Future Perspectives

**DOI:** 10.1155/2014/109786

**Published:** 2014-06-12

**Authors:** Mauro Pettorruso, Luisa De Risio, Giovanni Martinotti, Marco Di Nicola, Filippo Ruggeri, Gianluigi Conte, Massimo Di Giannantonio, Luigi Janiri

**Affiliations:** ^1^Institute of Psychiatry and Psychology, Catholic University of the Sacred Heart, Largo Agostino Gemelli 8, 00168 Rome, Italy; ^2^Department of Neuroscience and Imaging, “G. d'Annunzio” University, Chieti, Italy

## Abstract

Pathological gambling or gambling disorder has been defined by the DSM-5 as a behavioral addiction. To date, its pathophysiology is not completely understood and there is no FDA-approved treatment for gambling disorders. Glutamate is the principal excitatory neurotransmitter in the nervous system and it has been recently involved in the pathophysiology of addictive behaviors. In this paper, we review the current literature on a class of drugs that act as modulating glutamate system in PG. A total of 19 studies have been included, according to inclusion and exclusion criteria. Clinical trial and case series using glutamatergic drugs (N-acetylcysteine, memantine, amantadine, topiramate, acamprosate, baclofen, gabapentin, pregabalin, and modafinil) will be presented to elucidate the effectiveness on gambling behaviors and on the related clinical dimensions (craving, withdrawal, and cognitive symptoms) in PG patients. The results have been discussed to gain more insight in the pathophysiology and treatment of PG. In conclusion, manipulation of glutamatergic neurotransmission appears to be promising in developing improved therapeutic agents for the treatment of gambling disorders. Further studies are required. Finally, we propose future directions and challenges in this research area.

## 1. Background

Pathological gambling (PG) is characterized by persistent and maladaptive gambling behavior, whereby individuals engage in frequent and repeated episodes of gambling despite serious adverse consequences [1]. Gambling disorder affects 0.2–5.3% of adults worldwide; the devastating consequences of this behavioral disturbance often entail severe damage to the lives of patients and their families. To date, there is no FDA-approved treatment for PG, despite almost a decade of intense research, and effective treatment strategies remain very challenging. Recently, PG has been included in the diagnostic category of substance use and addictive disorders in the 5th edition of the Diagnostic and Statistical Manual of Mental Disorders (DSM-V).

Glutamate (Glu) is the principal excitatory neurotransmitter in the nervous system. It has been recently proposed that addiction can be viewed as the result of an impaired ability to inhibit drug seeking in response to environmental contingencies, due to alterations in Glu homeostasis, with combined activation of sensitized dopamine (DA) and N-methyl-d-aspartate (NMDA) glutamatergic receptors [2]. Blocking the release of Glu has prevented drug seeking behaviors in animals as well as patients with substance use disorders [3, 4]. The clinical and biological similarities between PG and drug addiction [5] suggest that PG patients may benefit from medication used to treat drug addiction and that pathophysiological models for drug addiction may be relevant to PG as well.

In this paper, we review the current literature on drugs that modulate glutamatergic neurotransmission in PG. We also elucidate current hypotheses on the neurobiology of PG, focusing on glutamatergic neurotransmission and its interactions with other neurotransmitters. Clinical trials and case series using glutamatergic drugs will be presented to elucidate the effectiveness on gambling behaviors and on the related clinical dimensions (craving, withdrawal, and cognitive symptoms) in PG patients. The results will be discussed to gain more insight into the pathophysiology and treatment of PG. Finally, we propose future directions and challenges in this research area.

## 2. Methods

Two reviewers were separately engaged in this review, following the same bibliographic search and data extraction protocol. Bibliographic search consisted of a computerized screening of Medline, Scopus, and Google Scholar database in January 2014. Only English language studies published in the last ten years were reviewed. We used the following queries: “gambl*” combined with “glutamate” and with a list of glutamatergic neurotransmission-modulating agents including N-acetylcysteine, memantine, amantadine, acamprosate, topiramate, lamotrigine, baclofen, gabapentin, pregabalin, modafinil, riluzole, dizocilpine, LY354740, D-cycloserine, methadone, and dextromethorphan. The search initially yielded 99 results. We then hand-searched relevant references of each article, including earlier studies on the topic.

**Figure 1:**
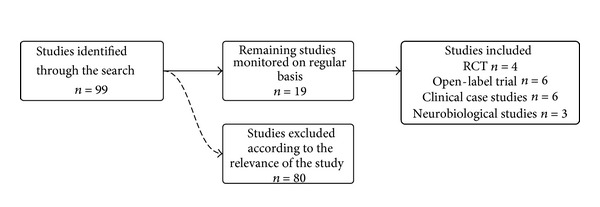
Bibliographic process.

Of the 99 potential articles, 19 were included (Figure 1) according to the following criteria: (a) the target problem is PG; (b) the abstract is available; (c) the publication is an original paper, excluding reviews; (d) the study is a neurobiological or a clinical research on PG subjects.

Table 1 shows relevant data from the articles included in the study: drug used, dosage, study design, sample size and targeted population, methods, cognitive outcome, and main finding on gambling outcome.

## 3. Glutamatergic Transmission in Addictive Behaviors: Relevance for Pathological Gambling

Glu is the most prevalent excitatory neurotransmitter in the CNS and its action is regulated by two types of receptors: the ionotropic (iGlu) and metabotropic (mGlu) receptors. The ionotropic receptors are ion channels that, upon Glu binding, increase the influx of sodium and potassium cations causing depolarization of the membrane [19]. They are divided into three subtypes: N-methyl-D-aspartate (NMDA), *α*-amino-3-hydroxy-5-methyl-4-isoazole-propionic acid (AMPA), and kainate. The metabotropic receptors are G protein-coupled receptors and are divided into three groups (I, II, and III) based on the homology of the sequences, the mechanism of signal transduction, and their pharmacological selectivity [20]. The metabotropic receptors are located primarily in the limbic and frontal areas, which are specifically involved in the mechanisms of addiction. In particular, receptors of group I seem to have an important role in the regulation of the reinforcing effects of drugs, while type II receptors are implicated in synaptic changes that occur as a result of prolonged exposure to the drug and in withdrawal syndromes [21]. Following abuse of any substance, increased glutamatergic transmission occurs in the limbic system and the prefrontal cortex which seems to be responsible, first and foremost, a greater release of DA, and also DA-dependent effects. In particular, while phenomena such as sensitization, craving, relapse, and reinforcement are linked to changes in both dopaminergic and glutamatergic systems, specific context and conditioned behaviors related to substance use primarily depend on glutamatergic mechanisms [22]. Summarily, the glutamatergic-dopaminergic system (in the nucleus accumbens) is responsible for the onset of “drug seeking,” while relapse only involves the glutamatergic system [23]. Reduction of extracellular glutamate levels in the limbic areas seems to be closely related to the withdrawal syndrome from psychostimulants; metabotropic glutamate receptor agonists seem to be able to reduce craving and prevent relapse via a compensation mechanism. Also, antagonists of metabotropic receptors hinder the behavioral effects of cocaine, nicotine, and alcohol, and NMDA antagonists are potential candidates for the treatment of opiate, alcohol, and sedative withdrawal syndromes [24].

PG has been presumed to be modulated mainly by brain DA and Glu, though findings are contrasting. DA is implicated in rewarding, reinforcing, and addictive behaviors. In drug addiction, data support the existence of a hypodopaminergic state at both the presynaptic and postsynaptic levels [25]. While DA release may reinforce learning [26, 27], Glu may be implicated in long-lasting neuroadaptations in the corticostriatal circuitry that represents the putative neural substrate of enduring vulnerability to relapse [2]. Glu is involved in learning and memory and may activate different types of Glu receptors, including NMDA receptors expressed in brain regions comprising reward circuitry [2]. Levels of Glu within the nucleus accumbens mediate reward-seeking behavior [2]. Pathological gamblers report euphoric feelings during gambling episodes, comparable to the “high” in substance use, thus making them more prone to continued gambling. In addition, preliminary reports showed a reduction of hedonic capacity in response to stimuli usually perceived as rewarding [28]. By continued gambling, the salience attribution to the behavior is strengthened and induces cue reactivity which can result in craving phenomena and potentially further enhancement of DA neurotransmission. Finally, continued gambling and subsequent altered DA neurotransmission could lead to neuroadaptation in mesolimbic-prefrontal glutamatergic pathways [29]. Chronic drug intake is associated with neuroadaptation of glutamatergic neurotransmission in the ventral striatum and limbic cortex [30]. In addition, cue exposure has been found to depend on strong projections of glutamatergic neurons from the prefrontal cortex to the nucleus accumbens [31]. Repetitive behaviors closely followed by rewards increase extracellular Glu levels [32]. In one study, cerebrospinal fluid (CSF) levels of glutamic and aspartic acid, both of which bind to NMDA receptors, were elevated among PG patients as compared to control subjects [33]. The imbalance in Glu homeostasis engenders changes in neuroplasticity which impair communication between the prefrontal cortex and the nucleus accumbens, thus favoring engagement in reward-seeking behaviors, such as PG [34].

## 4. Glutamatergic Treatment Strategies in Pathological Gambling

Manipulation of glutamatergic neurotransmission is a relatively young but promising avenue for the development of improved therapeutic agents for the treatment of drug and behavioral addictions [10, 35]. Substantial evidence has accumulated indicating that ligands acting on glutamatergic transmission are also of potential utility in the treatment of drug addiction, as well as various behavioral addictions such as pathological gambling. Growing evidence suggests that the glutamatergic system is central to the neurobiology and treatment of mood disorders [36] and that it could represent a valuable target in PG with comorbid conditions [37].

### 4.1. N-Acetylcysteine

N-Acetylcysteine (NAC), a cysteine prodrug and amino acid, can increase extracellular levels of Glu concentration in the nucleus accumbens and has shown preliminary efficacy in treating substance addictions [38, 39]. NAC may stimulate inhibitory metabotropic Glu receptors, possibly causing a reduction in synaptic release of glutamate. Studies in rat populations show that NAC is effective in reducing reward-seeking behavior [40] and preliminary data in PG are encouraging.

NAC was found to be effective in reducing gambling urges and behavior (lower scores on the Yale-Brown Obsessive Compulsive Scale modified for PG (PG-YBOCS)) in a small clinical trial [14]. Twenty-seven PG subjects (12 women) were treated for 8 weeks with NAC (mean dose was 1476.9 ± 311.3 mg/day). Responders were randomized in a 6-week double-blind discontinuation trial (NAC vs placebo). A significantly higher percentage of subjects treated with NAC still meet responder criteria at the end of the study (83.3% in NAC versus 28.6% in placebo group). In addition, a recent RCT confirmed the efficacy of NAC augmentation of behavioral therapy in the treatment of PG [15]. The study was conducted on 28 subjects with cooccurring nicotine dependence and PG. They received behavioral therapy and were randomized to augmentation with NAC (up to 3,000 mg/day) or placebo in a double-blind trial. During the final 3-month followup, there was a significant additional benefit for NAC versus placebo on gambling severity measures (PG-YBOCS).

Several matters remain unresolved. The optimal dose of NAC for PG is still unknown. The dose used in the augmentation-RCT was notably higher than that used in the previous study. According to preclinical data in rats, lower concentrations of NAC inhibit Glu transmission in the nucleus accumbens core while higher concentrations countermand this effect [41]. Given NAC glutamatergic properties and glutamate's role in learning and memory in addictive processes [42], its use has been proposed for patients who report craving to gamble and for those who are also undergoing an exposure-based psychosocial intervention.

### 4.2. Memantine

Memantine, a noncompetitive antagonist at the NMDA receptor with neuroprotective properties, is approved for Alzheimer's disease and is increasingly being studied in a variety of psychiatric disorders [43]. In PG patients memantine decreased PG-YBOCS scores and time spent gambling, also improving neurocognitive function related to cognitive flexibility [11]. Twenty-nine subjects were enrolled in a 10-week open-label trial. After memantine treatment (10–30 mg/day), PG-YBOCS scores and hours spent gambling decreased significantly. In addition, subjects underwent pre- and posttreatment cognitive assessment using the stop-signal task and the intradimensional/extradimensional (IDED) set shift task to assess impulsivity and cognitive flexibility, respectively. At study endpoint, a significant improvement in IDED performance was found, probably due to memantine modulation of glutamatergic transmission in PFC [44]. Nonetheless, the extent to which memantine exerts its influences on gambling behaviors through effects on impulsivity or compulsivity is still unclear [45].

A clinical case study reports effectiveness of memantine in the treatment of a 23-year-old patient with obsessive-compulsive disorder, body dysmorphic disorder, and severe PG [12]. A clinical response was observed after 8 weeks of memantine treatment, with more control over gambling and less anticipatory tension and excitation.

Memantine seems to reduce Glu excitability and improve impulsive decision-making. In addition, it shows promise in the treatment of cognitive and compulsive symptoms in PG patients [11, 45].

### 4.3. Amantadine

Amantadine, an antiglutamatergic drug with additional actions on dopaminergic neurotransmission, has been evaluated in treating PG and other compulsive behaviors in individuals with Parkinson's disease [9, 46]. Conflicting data have been reported regarding use of amantadine among Parkinson's disease patients [47]. It was found to be safe and effective in 17 patients with PG, reducing or stopping gambling urges and behaviors [9]. In a cross-sectional study amantadine was associated with PG and other impulse control disorders [48].

In addition, a case study suggested the possible utility in the treatment of PG patients [8]. A significant improvement on gambling symptoms suggests that simultaneous pharmacological modulation of the glutamatergic and dopaminergic systems may reduce gambling in PG, possibly reversing neuroplasticity-based pathological changes determined by addictive behaviors [2].

### 4.4. Topiramate

Topiramate is a glutamatergic antagonist and pro-GABAergic drug that significantly reduces impulsive behavior and compulsiveness. It has been tested and found to be effective versus placebo in disorders in which impulsivity and craving represent core features, such as alcohol dependence, cocaine dependence, bulimia nervosa, and binge eating disorder. In addition, it has recently been proposed that topiramate is also an antagonist of AMPA receptors, a Glu receptor subtype that mediates relapse-like behaviors and is implicated in the neuroadaptive changes produced by drugs of abuse as well [49].

A 14-week, randomized, double-blind, placebo-controlled trial investigated topiramate in PG [17]. Though no significant differences between the placebo group and the topiramate-treated group were observed with respect to primary outcome measures (change in the obsessions subscale of the PG-YBOCS), topiramate reduced impulsivity (particularly, motor and nonplanning impulsivity), as measured with the Barratt Impulsiveness Scale (BIS). The authors suggest that topiramate could be useful in PG subgroups characterized by high levels of impulsivity. Dannon et al. [16] compared the effectiveness of topiramate versus fluvoxamine in the treatment of PG in a 12-week, blind-rater comparison trial. Though the authors conclude that both topiramate and fluvoxamine monotherapies may be effective in the treatment of PG, improvement on the PG-CGI for fluvoxamine did not quite reach statistical significance. Also, a smaller number of dropouts were reported in the topiramate group.

In addition, in a patient with bipolar disorder and comorbid PG, Nicolato et al. [18] reported full remission of gambling craving and behavior after topiramate was added to standard lithium treatment.

### 4.5. Acamprosate

Acamprosate (calcium acetylhomotaurinate) is a taurine derivative and a nonspecific GABA agonist that promotes a balance between excitatory and inhibitory neurotransmitters (Glu and GABA). It binds specifically to GABAB receptors and appears to block Glu receptors and inhibit hyperactive glutamatergic signaling [50]. Although there is accumulated evidence suggesting that acamprosate interferes with the Glu system by antagonizing NMDA receptor activity [51], its mechanism of action still remains unclear. Recent findings suggest the involvement of calcium-mediated pathways [52]. These inconsistencies are perhaps related to factors such as brain region examined, NMDA receptor subunit composition, state of neuronal excitation, and the presence of various endogenous NMDA receptor neuromodulators such as polyamines [50, 53]. Acamprosate has been approved by the FDA for alcohol dependence. Restoring the imbalance between excitatory and inhibitory neurotransmissions caused by chronic alcohol exposure [53], it has been found to raise the continuous alcohol abstinence rate and double the days of cumulative abstinence from alcohol [54].

Contrasting results have been reported on its use in PG treatment [55]. In an 8-week, open-label trial following a 2-week observation, acamprosate significantly improved PG-YBOCS and Gambling Severity Assessment Scale (G-SAS) scores, both CGI scales, and number of gambling episodes [6]. Twenty-six patients received the medication (1,998 mg/day). The primary efficacy measure was the PG-YBOCS. Secondary efficacy measures included the G-SAS, the Clinical Global Impression (CGI) Improvement and Severity scales, a patient self-rated global rating, the Hamilton Depression Rating Scale (HDRS), the Sheehan Disability Scale (SDS), and the timeline follow back (TLFB).

In contrast, a parallel study failed to confirm its effectiveness on gambling behavior [7]. In this open-label study, 8 pathological gamblers treated with acamprosate 999 mg/day were evaluated monthly for 6 months to assess relapse. None of the patients attained 6 months of abstinence, defined as the absence of any gambling behavior during the month preceding the follow-up visit. VAS scores at baseline, after 1 month, and at relapse showed no statistically significant differences. No validated scales were employed to determine the effectiveness of acamprosate on gambling urges and craving.

### 4.6. Baclofen

Baclofen (beta-(4-chlorophenyl)-GABA) is a GABAB receptor agonist that has been found to suppress both acquisition of alcohol drinking behaviors in rats and daily alcohol intake in alcohol experienced rats. By inhibiting multivesicular release from the presynaptic terminal, it decreases synaptic Glu signaling [56] and inhibits Ca2+ permeability of NMDA receptors. In rats, it also suppresses alcohol-stimulated dopamine release in the shell of the nucleus accumbens [57].

In an open-label trial [7], 9 patients receiving baclofen were evaluated on a monthly basis in order to assess measures of sustained improvement (i.e., abstinence) and relapse. None of the patients attained 6 months of abstinence, defined as the absence of any gambling behavior during the month preceding the follow-up visit; only one patient receiving baclofen attained 4 months of abstinence. VAS scores at baseline, after 1 month, and at relapse showed no statistically significant differences.

### 4.7. Gabapentin and Pregabalin

Anticonvulsants, like gabapentin and pregabalin, have multiple mechanisms of action, including inhibition of presynaptic voltage-gated Na+ and Ca2+ channels, thereby inhibiting the relapse of neurotransmitters including glutamate. Gabapentin modulates both GABAergic and glutamatergic neurotransmissions. Several authors have explored the use of gabapentin in substance use disorders. Gabapentin reverses GABA deficits and Glu excess thought to underlie alcohol withdrawal and early abstinence. It reduces alcohol consumption and craving, thereby facilitating abstinence [58]. Pregabalin is a structural analog of GABA, similar to gabapentin. It also reduces excitatory neurotransmitter release and postsynaptic excitability. The FDA has approved pregabalin for partial epilepsy, neuropathic pain, and generalized anxiety disorders. In addition, pregabalin has been extensively studied in alcohol and benzodiazepine dependence [59]. A 6-month pilot trial preliminarily investigated the potential utility of their use in PG patients (6 patients received pregabalin; 4 patients received gabapentin), with a reduction of gambling craving as measured by G-SAS [10]. Also, pregabalin has been used to treat a case of citalopram-associated gambling onset [60]. Future studies should investigate the use of gabapentin and pregabalin in the treatment of PG, given that this drug seems to specifically target the central features of impulsivity, anxiety, and craving.

### 4.8. Modafinil

Modafinil is an atypical stimulant, originally designed to enhance wakefulness and vigilance in the treatment of narcolepsy and sometimes prescribed as an off-label treatment for attention-deficit/hyperactivity disorder (ADHD). Although its mechanisms of action are not completely understood, modafinil does not appear to act as a monoamine releaser as is the case for amphetamine-like stimulants. Rather, modafinil may act by stimulating *α*-adrenoceptors, suppressing GABA release, weakly inhibiting the dopamine transporter, or stimulating hypothalamic orexin-containing neurons [61, 62]. While most studies suggest a dopaminergic basis for its stimulant effects [63], modafinil has been shown to elevate extracellular levels of glutamate in numerous brain regions including the dorsal striatum, hippocampus, and diencephalon without affecting glutamate synthesis [35, 64]. Numerous clinical reports have shown that modafinil demonstrates potential efficacy in the treatment of cocaine addiction [62].

Zack and Poulos [13], in a placebo-controlled double-blind trial, tried to determine if modafinil (mean dose 200 mg/day) reduces the reinforcing effects of slot machine gambling in PG subjects and if this effect is stronger in high versus low impulsivity subjects (*N* = 20). Bet size declined uniformly in both high and low impulsivity participants taking modafinil. In high impulsivity participants, modafinil decreased desire to gamble, salience of gambling words, disinhibition, and risky decision-making. In low impulsivity participants, modafinil increased scores on these indices. The results showed that modafinil had bidirectional effects in the two groups. The same sample of patients was reevaluated in a prospective study, with clinical results highlighting that modafinil may discourage pathological gamblers from chasing losses but also encourage them to continue betting, rather than quitting while they are ahead [65]. Also, it has been reported a case of clear-cut temporal relationship between modafinil treatment and pathological gambling in a 39-year-old patient with a history of narcolepsy and associated cataplexy [66].

## 5. Discussion

There is substantial evidence indicating that pharmacological treatments targeting glutamatergic transmission are of potential utility in the treatment of drug addiction. Given that neurobiological findings indicate that PG and drug addiction share common etiopathological pathways [5, 45], drugs targeting glutamatergic transmission could be of use for the treatment of behavioral addictions (i.e., PG) as well.

The data seem to confirm the utility of targeting the glutamatergic system for the treatment of PG, in particular by acting on craving and increasing treatment retention [10, 15]. Glutamatergic medications may, in fact, offer some advantages in preventing relapse [4]. It has been recently proposed that addiction can be viewed as the result of an impaired ability to inhibit drug seeking in response to environmental contingencies, due to alterations in Glu homeostasis, with combined activation of sensitized DA and NMDA glutamatergic receptors [2]. Glutamatergic drugs may regulate the complex interactions between the glutamatergic and dopaminergic systems, acting simultaneously on both systems, in ways that need to be better explored.

Studies discussed are not homogeneous with respect to the criteria used to evaluate the effectiveness of pharmacological treatments for PG. In fact, some studies consider the absence of gambling behavior as the primary outcome while overlooking important clinical dimensions including craving and withdrawal symptoms. Interestingly, research on glutamatergic drugs highlights the importance of pointing clinical attention to the detection and treatment of cognitive symptoms [29]. Pathological gamblers exhibit a pattern of decision-making that repeatedly ignores long-term negative consequences in order to obtain immediate gratification or relief from uncomfortable states associated with their addiction. A variety of cognitive and emotional processes influence decision-making [11]. These alterations (i.e., cognitive inflexibility) may contribute to deviant choice in PG patients and to the maintenance of the disorder, as indirectly confirmed by the potential efficacy of cognitive therapy focused on altering irrational gambling cognition [67]. Targeting this clinical dimension, throughout the pharmacological modulation of the glutamatergic system, could be a useful treatment perspective and needs further study.

Drugs that enhance decision-making and executive function abilities are less well known because of the complexity of these functions which comprise different subprocesses (i.e., reward, punishment sensitivity, and impulsivity). However, it can be argued that agents targeting these subprocesses may improve decision-making as well. In addition, cognitive enhancers such as modafinil might also have beneficial effects, particularly in high impulsivity subjects [13].

## 6. Future Perspectives

**Figure 2:**
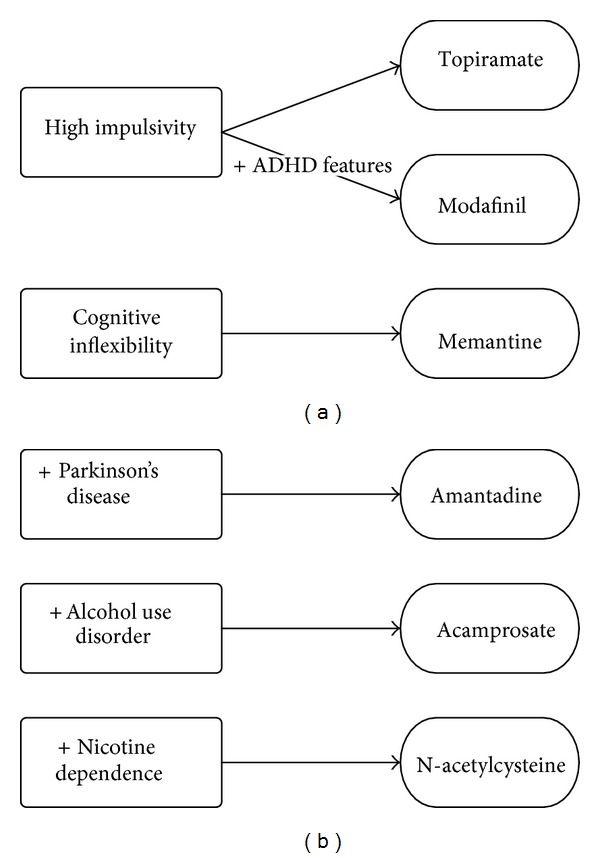
Clinical domains and comorbidity issues in the selection of glutamatergic treatment strategies to treat pathological gambling.

The data seem to confirm the utility of targeting the glutamatergic system for the treatment of PG, in particular by acting on craving and cognitive domains (impulsivity and cognitive inflexibility). While empirically validated treatments for PG have varying degrees of support, little is known about their mechanisms of action or how specific therapies might work better for specific individuals. Several studies have been conducted to test the efficacy of opioid antagonists in the treatment of the disorder, and a genetic predisposition or a family history of alcoholism has been hypothesized to regulate response to opioid antagonists across diagnostic groups [68]. Similarly, future studies should investigate the biological and psychological features of PG patients for whom glutamatergic treatment is appropriate. Based on current knowledge, we suggest clinical domains and comorbidity issues that may help guide the clinicians in the selection of appropriate glutamatergic treatment strategies (Figure 2). This model may provide the basis and rationale to guide the selection of pharmacotherapies in some groups of PG patients. Further investigations are certainly needed to confirm the treatment algorithm we propose.

Following cocaine administration, disrupted Glu homeostasis of nucleus accumbens core has been observed. A hallmark of disrupted homeostasis is a decrease in expression and function of the major Glu transporter, GLT-1 [69]. Future studies should investigate its role in PG and the potential utility of drugs that act to modulate the expression of Glu neurotransmitter transporters via gene activation (i.e., ceftriaxone) [70].

Besides Glu and DA, other factors, like brain-derived neurotrophic factor (BDNF), can be involved in the action of glutamatergic agents in PG [71]. Neurotrophic factors have been shown to be modulated by environmental events in various psychopathological conditions [72], and their role has been confirmed in the pathophysiology of PG [73]. Future studies should help understand the potential role of glutamatergic modulation on neurotrophins levels in PG patients.

Future investigations would benefit from placebo-controlled clinical trials to outline the true benefits of glutamatergic drugs for the treatment of PG. In addition, future research could profit from pharmacological challenges in combination with neuroimaging techniques to shed light on Glu role in the pathophysiology of PG. New neurobiological PG research should include matched controls, account for comorbidity issues, and differentiate between gambling preferences. Investigations in specific subgroups, therefore, are expected to give more insight into the pathophysiology of the disorder in these groups and perhaps lead to more tailored and efficient therapies. Future studies should also focus on the functional connections between dopaminergic and glutamatergic systems, in order to shed light upon the complex neurobiological mechanisms underlying the development of maladaptive gambling behavior.

## Figures and Tables

**Table 1:** Clinical trials and case series using glutamatergic drugs to treat pathological gambling.

Drug	Dosage (mg/day)	Study design	Duration	Sample size	Methods	Cognitive outcome	Gambling outcome	Comments	References
Acamprosate	1,998	Open-label	8 + 2 weeks	26 PG pts	PG-YBOCS, G-SAS, CGI, gambling episodes	NA	77% of participants completed. Improvement on all efficacy scales. 65% were responders.	Improvement in ADHD Checklist scores.	Black et al., 2011 [6]
	999	Open-label	6 months	8 PG pts	Gambling relapse, VAS	NA	None reached 6-month abstinence. No change in VAS scores to relapse.		Dannon et al., 2011 [7]

Amantadine	150	Clinical case study	8 weeks	One PG patient	G-SAS, HDRS, YMRS	NA	Reduction of 43–64% in gambling symptoms severity (G-SAS).		Pettorruso et al., 2012 [8]
	200	Double-blind, placebo-controlled	17 weeks	17 PG pts with Parkinson's disease	G-SAS, PG-YBOCS, SOGS	NA	Abolished daily PG in 7 pts. 5 pts reduced daily expenditures and time spent gambling.	Valuable option in Parkinson's disease gambling behaviors	Thomas et al., 2010 [9]

Baclofen	30–50	Open-label	6 months	9 PG pts	Gambling relapse, VAS	NA	One patient reached 4-month abstinence. None reached 6-month abstinence. No change in VAS scores to relapse.		Dannon et al., 2011 [7]

Gabapentin, pregabalin	300–600	Case series	6 months	GB: 4 PG pts PGB: 6 PG pts	G-SAS, VAS	NA		Reduction of gambling craving and withdrawal	Pettorruso et al., 2013 [10]

Memantine	10–30	Open-label	10 weeks	29 PG pts	PG-YBOCS, stop-signal task, IDED task	Reduced cognitive inflexibility	PG-YBOCS score and hours spent gambling decreased.	Target both gambling and cognitive deficits in PG	Grant et al., 2010 [11]
	20	Case report	8 weeks	One OCD, BD, PG pt	G-SAS		Reduction of more than 50% in GSAS scores.		Pavlovic, 2011 [12]

Modafinil	200	Double-blind, placebo-controlled	Single session	20 nontreatment-seeking PG pts	EIQ, IGT, cognitive tasks	In H-I pts reduced disinhibition and risky decision-making	In H-I pts decreased desire to gamble, salience, disinhibition, and risky decision-making. In L-I pts increased scores.	Impulsivity could moderate medication response in PG	Zack and Poulos, 2009 [13]

NAC	1,200–1,800	Open-label, double-blind discontinuation phase	8 + 6 weeks	27 PG pts	PG-YBOCS, G-SAS, CGI	NA	59.3% were respondents. Difference with placebo in discontinuation phase.	NAC targets craving in PG-addictive subtype	Grant et al., 2007 [14]
	1,200–3,000	RCT	12 weeks	28 PG, nicotine dependent, pts	SCI-PG, PG-YBOCS Fagerström test for nicotine dependence	NA	During the 3-month followup, NAC was superior to placebo on PG severity.	NAC facilitates long-term behavioral therapy	Grant et al., 2014 [15]

Topiramate	200	Randomized, blind-rater vs fluvoxamine	12 weeks	15 PG pts (topiramate), 16 PG pts (fluvoxamine)	SOGS, PG-YBOCS, CGI	NA	9/12 pts reported full remission and 3/12 partial remission. Significant CGI improvement.		Dannon et al., 2005 [16]
	300	RCT	14 weeks	20 PG pts 22 placebo	PG-YBOCS, G-SAS, CGI-I, BIS-11	Reduced impulsivity traits	No significant effect on the primary measures.	Small sample size. Study probably underpowered	Berlin et al., 2013 [17]
	200	Clinical case study (add-on to lithium)	2 months	One pt with BD and PG comorbidities	None	NA	Gambling behavior abated after 2 months of combined treatment. On long-term followup the patient remained asymptomatic.	Valuable add-on treatment in BD-PG comorbidity	Nicolato et al., 2007 [18]

RCT: randomized-controlled trial; PG: pathological gambling; G-SAS: Gambling-Symptom Assessment Scale; PG-YBOCS: Yale-Brown Obsessive-Compulsive Scale modified for pathological gambling; HDRS: Hamilton Depression Rating Scale; YMRS: Young Mania Rating Scale; NA: not available; IDED task: intradimensional/extradimensional set shift task; SOSG: South Oaks Gambling Screen; EIQ: Eysenck Impulsiveness Questionnaire; IGT: Iowa gambling task; OCD: obsessive compulsive disorder; BD: bipolar disorder; H-I: high impulsivity; L-I: low impulsivity.

## Abbreviations

PG: Pathological gambling
Glu: Glutamate
DA: Dopamine
NMDA: N-methyl-d-aspartate
AMPA: *α*-amino-3-hydroxy-5-methyl-4-isoazole- propionic acid
GABA: Gamma-aminobutyric acid
CSF: Cerebrospinal fluid
NAC: N-acetylcysteine
RCT: Randomized-controlled trial
PG-YBOCS: Yale-Brown Obsessive Compulsive Scale modified for PG
G-SAS: Gambling Severity Assessment Scale.
